# Identification of genes of prognostic value in the ccRCC microenvironment from TCGA database

**DOI:** 10.1002/mgg3.1159

**Published:** 2020-02-03

**Authors:** Bangbei Wan, Bo Liu, Yuan Huang, Cai Lv

**Affiliations:** ^1^ Department of Urology Central South University Xiangya School of Medicine Affiliated Haikou Hospital Haikou Hainan China; ^2^ Laboratory of Developmental Cell Biology and Disease School of Ophthalmology and Optometry and Eye Hospital Wenzhou Medical University Wenzhou Zhejiang China; ^3^ Department of Neurology Central South University Xiangya School of Medicine Affiliated Haikou Hospital Haikou Hainan China

**Keywords:** ccRCC, immune scores, microenvironment, stromal scores, TCGA database

## Abstract

**Background:**

Clear cell renal cell carcinoma (ccRCC) is the most common pathological subtype of renal cell carcinoma. Bioinformatics analyses were used to screen candidate genes associated with the prognosis and microenvironment of ccRCC and elucidate the underlying molecular mechanisms of action.

**Methods:**

The gene expression profiles and clinical data of ccRCC patients were downloaded from The Cancer Genome Atlas database. The ESTIMATE algorithm was used to compute the immune and stromal scores of patients. Based on the median immune/stromal scores, all patients were sorted into low‐ and high‐immune/stromal score groups. Differentially expressed genes (DEGs) were extracted from high‐ versus low‐immune/stromal score groups and were described using functional annotations and protein‒protein interaction (PPI) network.

**Results:**

Patients in the high‐immune/stromal score group had poorer survival outcome. In total, 95 DEGs (48 upregulated and 47 downregulated genes) were screened from the gene expression profiles of patients with high immune and stromal scores. The genes were primarily involved in six signaling pathways. Among the 95 DEGs, 43 were markedly related to overall survival of patients. The PPI network identified the top 10 hub genes—*CD19, CD79A*, *IL10*, *IGLL5*, *POU2AF1*, *CCL19*, *AMBP*, *CCL18*, *CCL21,* and *IGJ—*and four modules. Enrichment analyses revealed that the genes in the most important module were involved in the B‐cell receptor signaling pathway.

**Conclusion:**

This study mainly revealed the relationship between the ccRCC microenvironment and prognosis of patients. These results also increase the understanding of how gene expression patterns can impact the prognosis and development of ccRCC by modulating the tumor microenvironment. The results could contribute to the search for ccRCC biomarkers and therapeutic targets.

## Graphical abstract

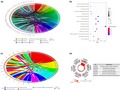

## 1 INTRODUCTION

Renal cell carcinoma (RCC) is a common malignant tumor of the urinary system (Jonasch et al., 2012), and clear cell renal cell carcinoma (ccRCC) is its most common pathological subtype. The morbidity and mortality of ccRCC are reportedly increasing every year (Motzer et al., 2017). Although the treatment of ccRCC has improved, the prognosis of ccRCC remains poor, especially in cases of locally advanced and metastatic ccRCCs (Dutcher, 2013). Therefore, the exploration of biomarkers associated with ccRCC diagnosis, therapy, and patient prognosis has become an important issue (Adashek, Salgia, Posadas, Figlin, & Gong, 2019).

The tumor microenvironment (TM) plays a vital role in the prognosis of patients with cancer. Components of the TM are very complicated. It primarily consists of nontumor components that include stromal cells and tumor cells, and tumor components that are predominantly tumor cells, stromal cells, and tumor cells. Nontumor components can be regarded as valuable indexes for the therapeutic and prognostic assessment of tumors. Wu and Dai (2017) reported that the TM noticeably affects the therapeutic response and clinical outcome, and can mediate drug resistance by inducing the secretion of soluble factors from tumor or stromal cells. Moreover, the adhesion of tumor cells to stromal fibroblasts or components of the extracellular matrix can also attenuate therapeutic responses. Şenbabaoğlu et al found that a decrease in immune cell content (such as Th17 cells) correlated with ccRCC progression and poor patient prognosis (Şenbabaoğlu et al., 2016). Ghatalia et al reported that sunitinib therapy significantly prolonged the disease‐free survival of the patients with high CD8^+^ T‐cell density (George et al., 2018). Although these studies explored the role of the TM in the development and prognosis of ccRCC, most have only highlighted the association between the TM components and patient prognosis. Few studies have used expression profile data from high‐throughput sequencing to examine the relationship between gene expression patterns in the TM and ccRCC prognosis.

In this study, we aimed to identify genes of prognostic value in the ccRCC microenvironment and examine the possible mechanisms underlying ccRCC development. We downloaded gene expression profiles and clinical information of ccRCC patients from The Cancer Genome Atlas (TCGA) and utilized the R programming language software to analyze the data for differentially expressed genes (DEGs). We used the DAVID database to conduct gene ontology (GO) functions and the Kyoto Encyclopedia of Genes and Genomes (KEGG) for pathway enrichment analyses of the DEGs. A protein‒protein interaction (PPI) network was constructed using the Search Tool for the Retrieval of Interacting Genes (STRING) online database and the results were downloaded through Cytoscape. Finally, we identified hub genes and constructed modules from the PPI network. The results of this study reveal reliable prognostic biomarkers and therapeutic targets for ccRCC.

## 2 MATERIALS AND METHODS

### 2.1 Database

The gene expression profiles and clinical data (e.g., gender, age, stage, grade, survival, and outcome) of patients with ccRCC were obtained from TCGA database (https://portal.gdc.cancer.gov/). Immune scores and stromal scores of ccRCC patients were calculated by applying the ESTIMATE algorithm using the estimate package in R (https://www.r-project.org/). All patients were divided into low‐ and high‐ immune/stromal score groups according to the median immune/stromal scores.

### 2.2 Identification of DEGs

We utilized the limma R package to identify the differentially expressed genes (DEGs) according to the following cut‐off value: False discovery rate (FDR) <0.05 and |log2 fold change (FC)| > 1.

### 2.3 GO and KEGG pathway enrichment analyses of DEGs

The DAVID 6.8 database (https://david.ncifcrf.gov/) is a widely used database for gene enrichment and functional annotation analyses. Using DAVID, we applied the GO function and KEGG pathway enrichment analyses to the identified DEGs, with a cut‐off criterion of *p*‐value <.05.

### 2.4 PPI network construction and analysis of modules

To help us understand the interactions between different genes, we used the STRING (https://string-db.org/) online database (Franceschini et al., 2013) to analyze the protein‒protein interaction (PPI) network of the DEGs. To identify modules in the PPI network, we used the Molecular Complex Detection (MCODE) plug‐in in the Cytoscape software (Shannon et al., 2003), with the following default parameters: “Degree Cutoff = 2,” “Node Score Cutoff = 0.2,” “K‐Core = 2,” and “Max.Depth = 100” (Bader & Hogue, 2003).

### 2.5 Identification and analysis of hub genes

The top 10 hub genes were selected using the degree algorithm (Chin et al., 2014) in cytoHubba (a Cytoscape plugin). The cBioPortal database online portal (http://www.cbioportal.org) was used to analyze the network of hub genes and their coexpressed genes.

### 2.6 Overall survival analysis

The survival R package was used to analyze the relationship between DEGs expression levels (including hub genes) and the overall survival of patients. We tested this relationship with a log‐rank test and landmark analysis, where *p* < .05 was regarded as statistically significant.

## 3 RESULTS

### 3.1 Immune scores and stromal scores are significantly correlated with clinical parameters

**Figure 1:**
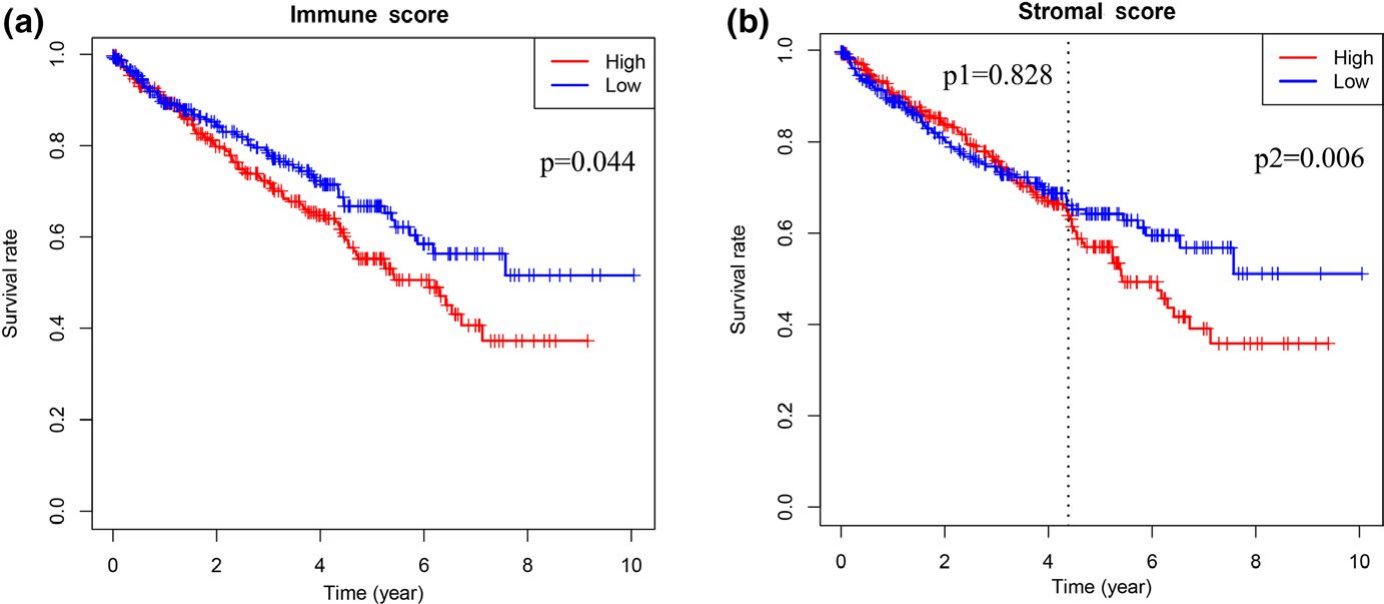
The correlation between (a) immune scores or (b) stromal scores of patients and overall survival. Immune scores and stromal scores were significantly associated with the prognosis of patients (*p* < .05)

**Figure 2:**
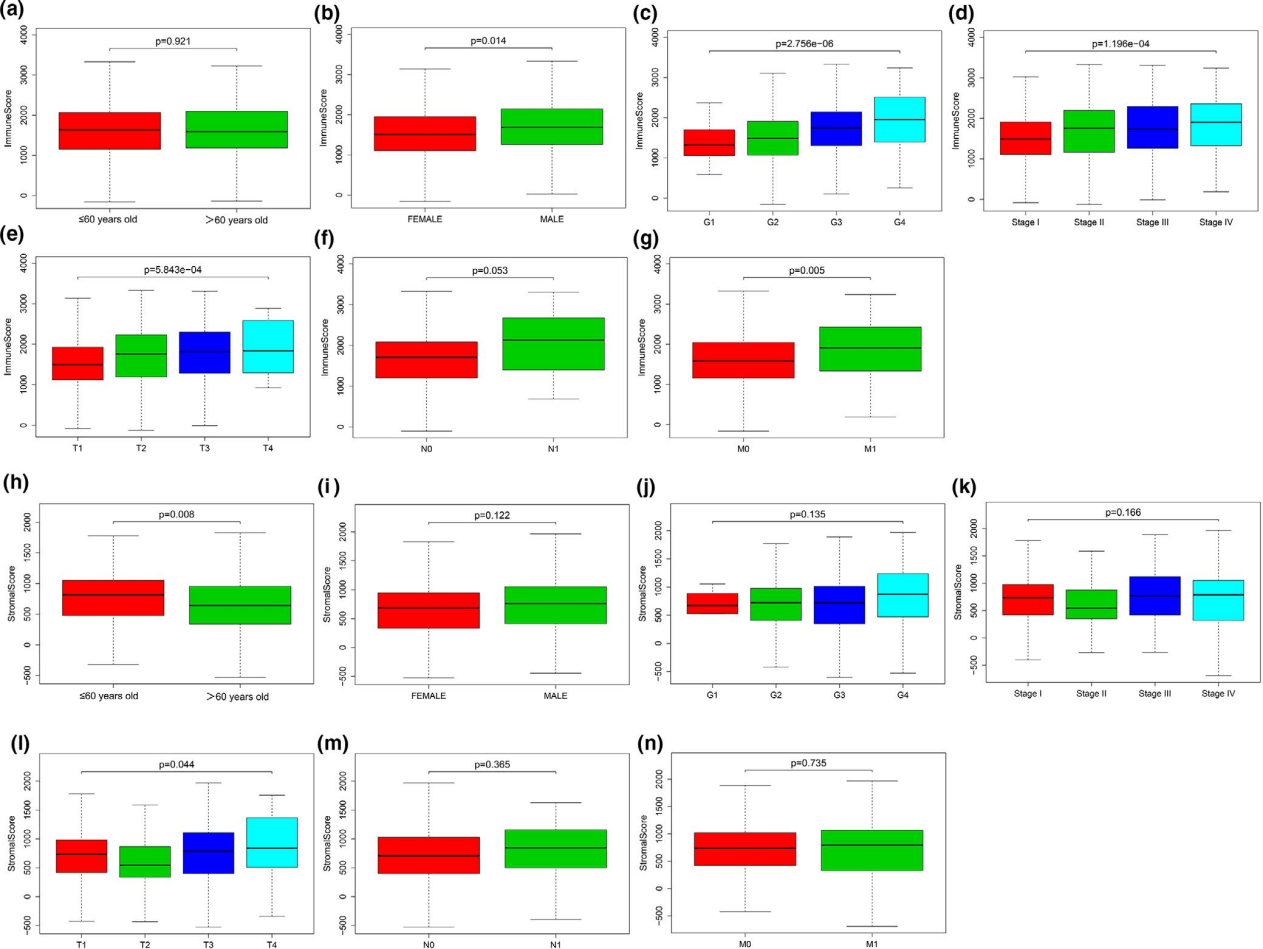
The relationship between immune scores or stromal scores of patients and clinical parameters. Immune scores were differential in gender, grade, clinical stage, T stage, and M stage parameters (*p* < .05); stromal scores were only differential in age and T stage parameters (*p* < .05). (a) Immune scores and age; (b) Immune scores and gender (*p* < .05); (c) Immune scores and grade (*p* < .05); (d) Immune scores and pathological stage (*p* < .05); (e) Immune scores and T stage (*p* < .05); (f) Immune scores and N stage; (g) Immune scores and M stage (*p* < .05); (h) Stromal scores and age (*p* < .05); (i) Stromal scores and gender; (j) Stromal scores and grade; (k) Stromal scores and pathological stage; (l) Stromal scores and T stage (*p* < .05); (m) Stromal scores and N stage; (n) Stromal and M stage

We downloaded gene expression profiles and clinical information of ccRCC patients from TCGA database. We then matched immune and stromal scores of these patients via the estimate package in R. Next, we obtained 530 patients with complete immune and stromal scores; stromal scores ranged from −1,433.76 to 1,967.19 and immune scores ranged from −693.95 to 3,328.20 (Table 1). The median immune/stromal score was used as the cut‐off value to classify all ccRCC patients into low‐ and high‐ immune/stromal score groups. Analyses of the relationship between patient immune or stromal scores and overall survival using the survival package in R showed that patients in the high‐immune score group had a poorer prognosis than those in the low‐immune score group. After 4.3 years of follow‐up, patients in the high‐stromal score group had a worse prognosis than those in the low‐stromal score group (Figure 1). In addition, we analyzed the relationship between patient immune or stromal scores and clinical parameters (Figure 2) and found that the clinical parameters (histological grade, pathological, T, and M stages) increased with higher immune scores (*p* < .05). Increased stromal scores were associated with an increase in T stage (*p* < .05).

**Table 1:** Immune scores, stromal scores, and clinical data of patients with ccRCC

Characteristic	*n*	Stromal score (range)	Immune score (range)
Age			
≤60	264	−1433.76 to 1778.24	−693.95 to 3,328.20
＞60	266	−1375.62 to 1967.19	−389.80 to 3,223.9
Gender			
Female	186	−1433.76 to 1828.05	−693.95 to 3,238.35
Male	344	−1413.81 to 1967.19	−660.28 to 3,328.2
Histological grade			
G1	14	−615.34 to 1,054.71	−136.19 to 2,368.85
G2	227	−1375.62 to 1768.6	−389.80 to 3,102.67
G3	206	−1433.76 to 1888.47	−693.95 to 3,328.2
G4	75	−690.60 to 1967.19	255.63 to 3,238.35
GX	5	−1413.81 to 945.64	−660.28 to 1859.09
Unknown	3	−385.32 to 654.11	362.53 to 1518.59
Stage			
I	265	−1433.76 to 1778.24	−693.95 to 3,135.31
II	57	−1413.81 to 1764.27	−660.28 to 3,328.2
III	123	−827.01 to 1888.47	−9.16 to 3,306.6
IV	82	−690.60 to 1967.19	189.47 to 3,238.35
unknown	3	822.33 to 1,346.92	780.29 to 3,094.80
T stage			
T1	271	−1433.76 to 1778.24	−693.95 to 3,135.31
T2	69	−1413.81 to 1764.27	−660.28 to 3,328.20
T3	179	−827.01 to 1967.19	−9.16 to 3,306.60
T4	11	−339.67 to 1757.07	925.24 to 2,884.05
N stage			
N0	239	−1413.81 to 1967.19	−660.28 to 3,328.20
N1	16	−394.15 to 1627.16	688.28 to 3,306.60
Nx	275	−1433.76 to 1778.24	−693.95 to 3,238.35
M stage			
M0	420	−1433.76 to 1888.47	−693.95 to 3,328.20
M1	78	−690.60 to 1967.19	189.47 to 3,238.35
Mx	30	−615.34 to 1,375.84	−136.19 to 3,022.26
Unknown	2	818.41 to 1752.31	1,424.61 to 2,235.77
Survival status			
Death	166	−1413.81 to 1967.19	−660.28 to 3,328.20
Alive	364	−1433.76 to 1757.07	−693.95 to 3,306.60

### 3.2 Identification of DEGs

**Figure 3:**
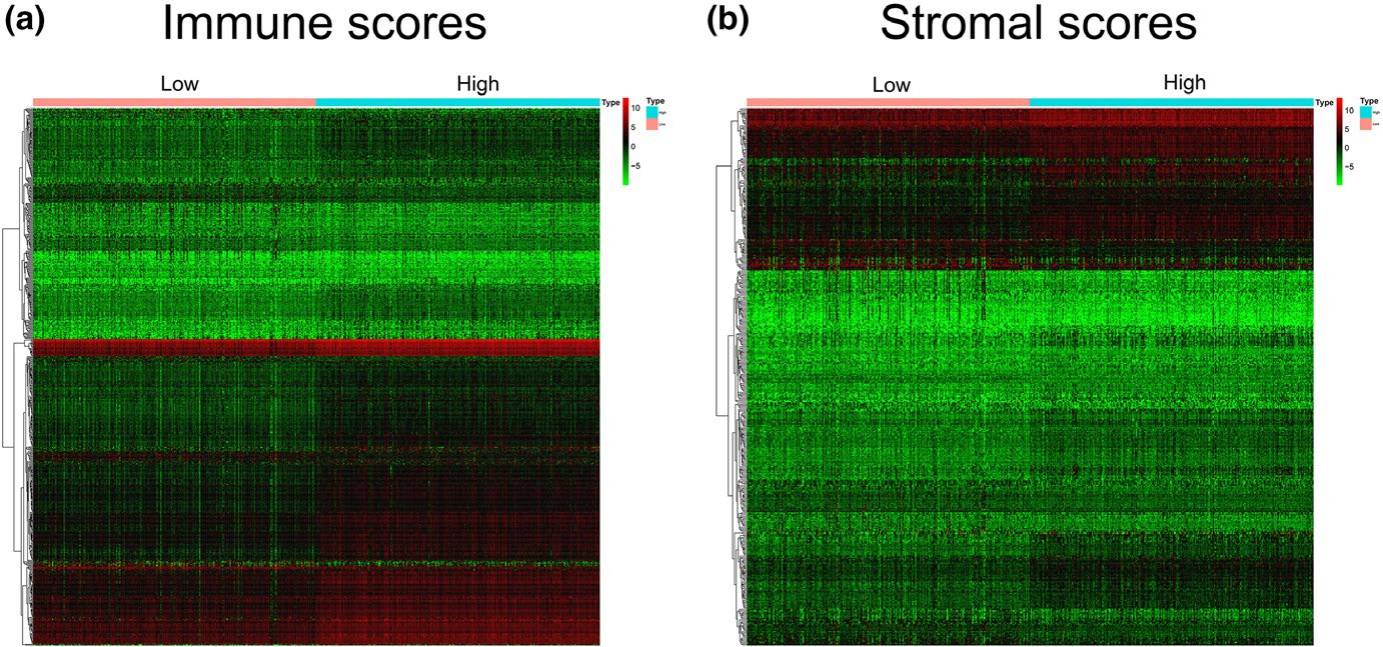
Heatmap of differential gene expression in the low score group (immune scores or stromal scores) and the high score group (immune scores or stromal scores). (a) Immune scores (low score in left and high score in right; |log2 fold change (FC)|> 1, FDR < 0.05); (b) Stromal scores (low score in left and high score in right; |log2 fold change (FC)|> 1, FDR < 0.05)

**Figure 4:**
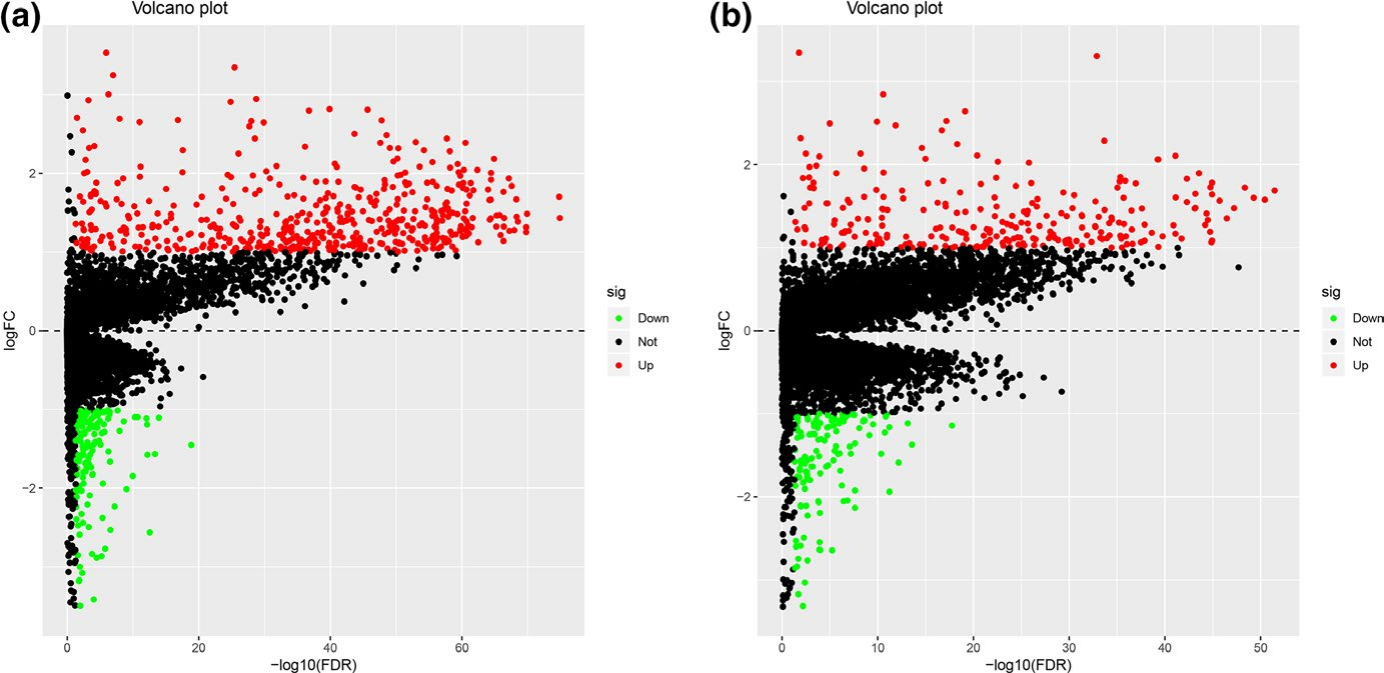
Differentially expressed genes (DEGs) in (a) immune scores and (b) stromal scores. Red represents upregulated genes, green represents downregulated genes, according to |log2 fold change (FC)|> 1, FDR <0.05

**Figure 5:**
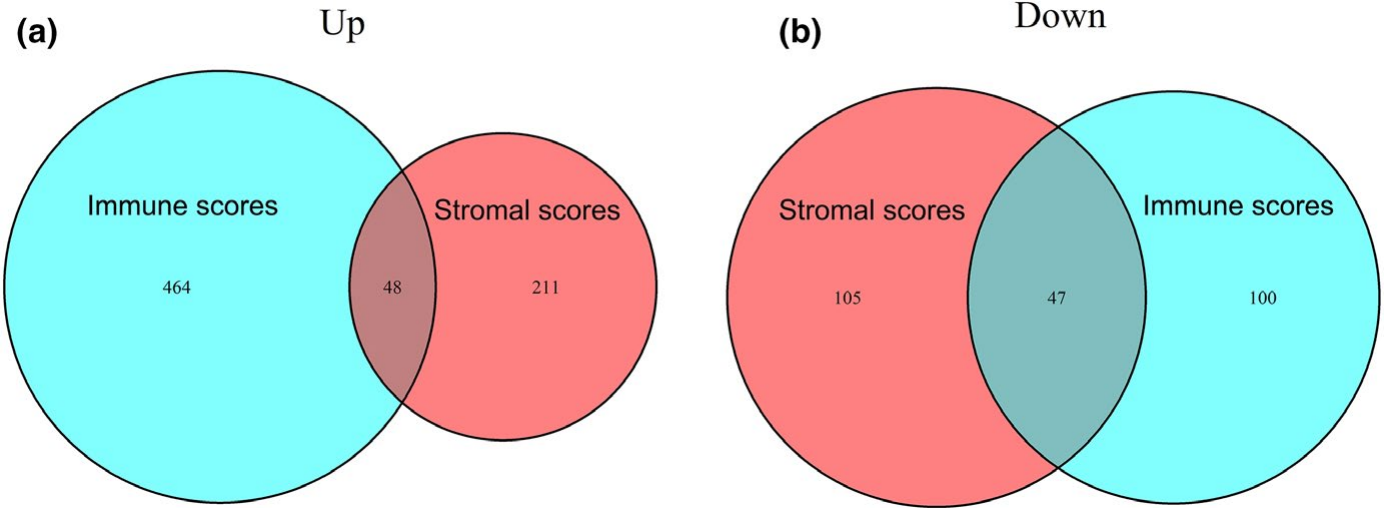
Common differentially expressed genes in immune scores and stromal scores. (a) Common upregulated genes; (b) common downregulated genes

To better understand the correlation between gene expression profiles and immune and/or stromal scores, we analyzed the gene expression profiles of the 530 ccRCC patients. We categorized the patients based on the median of their immune or stromal scores (high vs. low, for each); Figure 3 shows that the gene expression profiles can be used to differentiate the two groups. For immune scores, 512 genes were upregulated and 147 genes were downregulated in the high‐score versus low‐score group (|log2 fold change (FC)|> 1, FDR < .05; Figure 4a). Similarly, for stromal scores, 259 genes were upregulated and 152 genes downregulated in the high‐score versus low‐score group (|log2 fold change (FC)|> 1, FDR <0.05; Figure 4b). We also analyzed the shared upregulated and downregulated genes in both high‐score groups (immune and stromal scores) and found 48 upregulated and 47 downregulated genes (Figure 5). These 95 genes obtained by comparing the high versus low immune scores and stromal score groups were regarded as DEGs and subsequently used in further analyses.

### 3.3 Correlation between DEGs and overall survival

**Figure 6:**
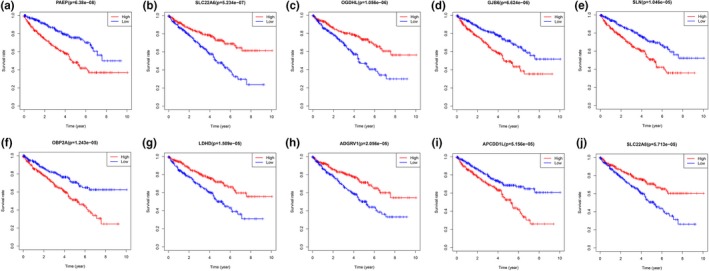
Top ten Kaplan–Meier analysis results of DEGs correlated with overall survival

To assess the prognostic value of the 95 DEGs, we analyzed the relationship between the expression of each DEG and overall survival of the ccRCC patients. Among the 95 DEGs, 43 were significantly related to overall patient survival (*p* < .05); the top 10 results are shown in Figure 6.

### 3.4 GO function and KEGG pathway enrichment analyses of DEGs

**Figure 7:**
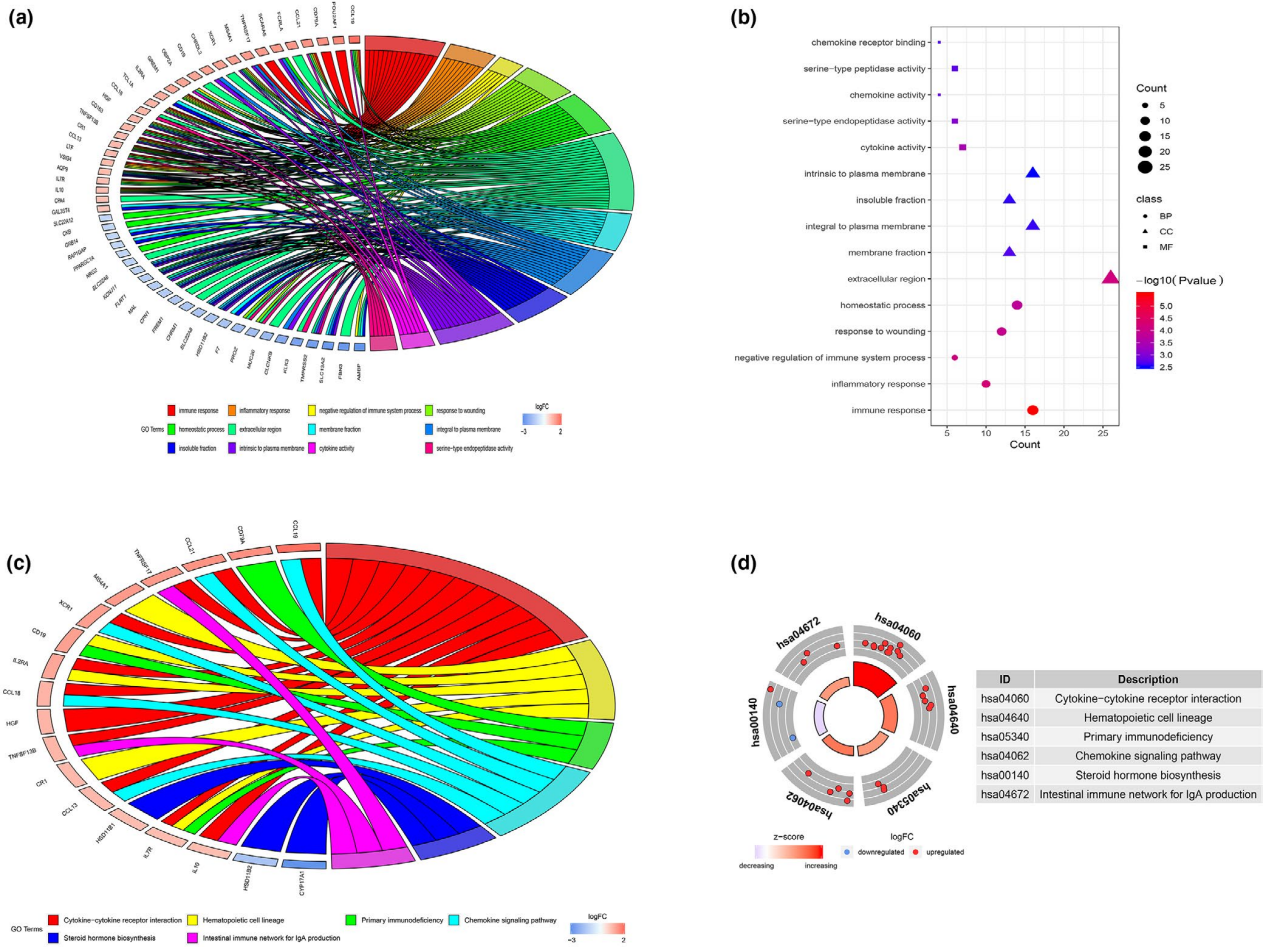
Top 15 GO enrichment terms of DEGs (a and b) and KEGG pathway analysis of DEGs (c and d)

To understand the potential mechanisms underlying the 95 DEGs and ccRCC development, we performed GO function annotation and KEGG pathway enrichment analyses using the DAVID database and its online analysis tool. The GO function analyses of the DEGs were divided into the following three parts: biological process (BP), molecular function (MF), and cell component (CC), and the top 15 results are shown in Figure 7a,b and Table 2. The DEGs were primarily enriched in the immune response (BP), extracellular region (CC), and cytokine activity (MF). Results of KEGG pathway enrichment analyses indicated that the DEGs were mainly enriched in six pathways involved in cytokine‒cytokine receptor interaction, hematopoietic cell lineage, primary immunodeficiency, chemokine signaling pathway, steroid hormone biosynthesis, and intestinal immune network for IgA production (Figure 7c,d and Table 3).

**Table 2:** Top 15 GO enrichment terms from analysis of DEGs

Category	ID	Term	Count	*p*‐value
BP	GO:0006955	Immune response	16	3.24105E‐06
BP	GO:0006954	Inflammatory response	10	5.99498E‐05
BP	GO:0002683	Negative regulation of immune system process	6	8.33701E‐05
BP	GO:0009611	Response to wounding	12	0.000118248
BP	GO:0042592	Homeostatic process	14	0.000167634
CC	GO:0005576	Extracellular region	26	7.3887E‐05
CC	GO:0005624	Membrane fraction	13	0.001961813
CC	GO:0005887	Integral to plasma membrane	16	0.002557573
CC	GO:0005626	Insoluble fraction	13	0.002656894
CC	GO:0031226	Intrinsic to plasma membrane	16	0.00317684
MF	GO:0005125	Cytokine activity	7	0.000354613
MF	GO:0004252	Serine‐type endopeptidase activity	6	0.000888895
MF	GO:0008009	Chemokine activity	4	0.001424593
MF	GO:0008236	Serine‐type peptidase activity	6	0.001692938
MF	GO:0042379	Chemokine receptor binding	4	0.001711249

**Table 3:** KEGG pathway analysis of DEGs

ID	Pathway	Count	*p*‐value	Genes
hsa04060	Cytokine‒cytokine receptor interaction	11	4.85E‐06	*CCL13 IL2RA TNFSF13B CCL21 CCL19 TNFRSF17 HGF IL7R XCR1 IL10 CCL18*
hsa04640	Hematopoietic cell lineage	5	0.002402127	*CR1 CD19 IL2RA MS4A1 IL7R*
hsa05340	Primary immunodeficiency	3	0.022496299	*CD19 CD79A IL7R*
hsa04062	Chemokine signaling pathway	5	0.034718017	*CCL13 CCL21 CCL19 XCR1 CCL18*
hsa00140	Steroid hormone biosynthesis	3	0.037388975	*CYP17A1 HSD11B1 HSD11B2*
hsa04672	Intestinal immune network for IgA production	3	0.041959354	*TNFSF13B TNFRSF17 IL10*

### 3.5 Construction of the PPI network of DEGs and analyses of the modules

**Figure 8:**
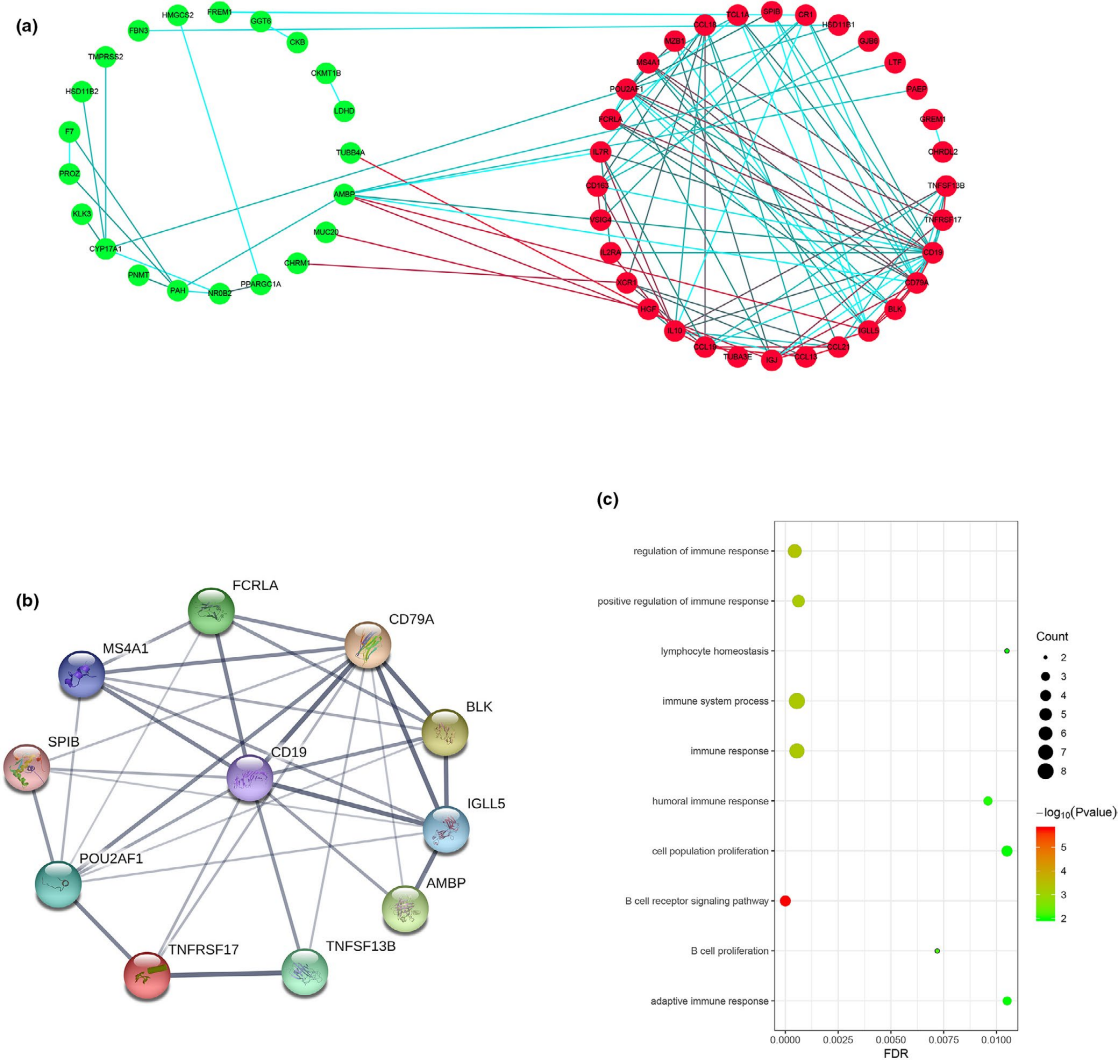
Constructed PPI network of DEGs and analysis of module. (a) PPI network of DEGs; red nodes represent upregulated DEGs and green nodes represent downregulated DEGs. The blue and red lines indicate the combined score from low to high. (b) The most important module; (c) biological process of all genes in the most important module

To further explore the interaction of the DEGs and their mechanisms underlying the regulation of ccRCC development, we utilized the STRING online database to analyze the DEGs and construct a PPI network. The results were downloaded and analyzed in the Cytoscape software. In the PPI network, we identified 53 genes (Figure 8a) and 4 function modules (using MCODE). We selected the most important module (Figure 8b) and used the STRING database to analyze the biological processes associated with the genes in this module. Our results indicate that the genes were mainly enriched in the B‐cell receptor signaling pathway (Figure 8c).

### 3.6 Identification and analyses of hub genes

**Figure 9:**
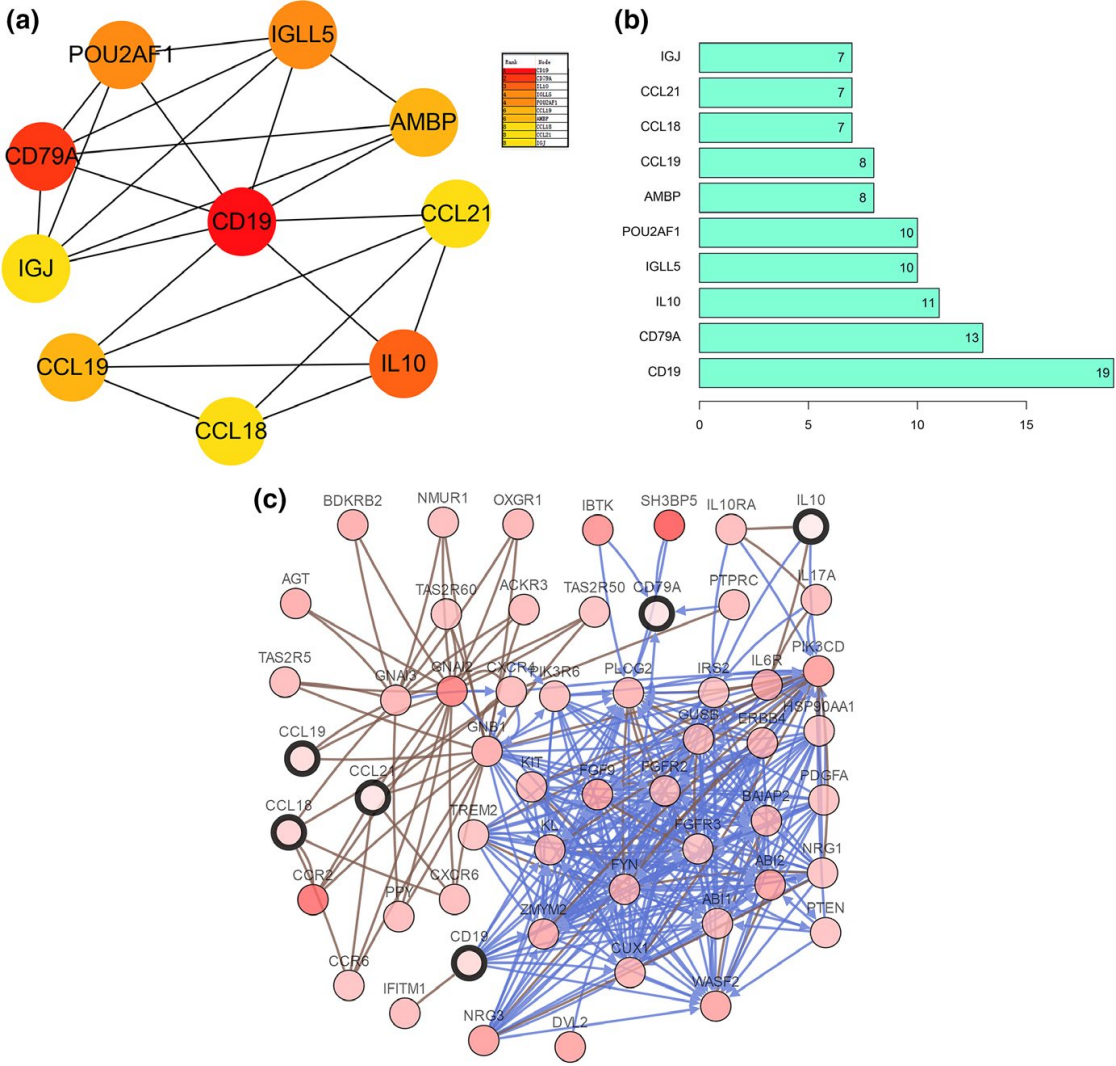
Identification and analysis of hub genes. (a) The ten hub genes were identified using Cytoscape. (b) Degree value of the ten hub genes; (c) hub genes and their co‐expression genes were analyzed via cBioPortal database

**Figure 10:**
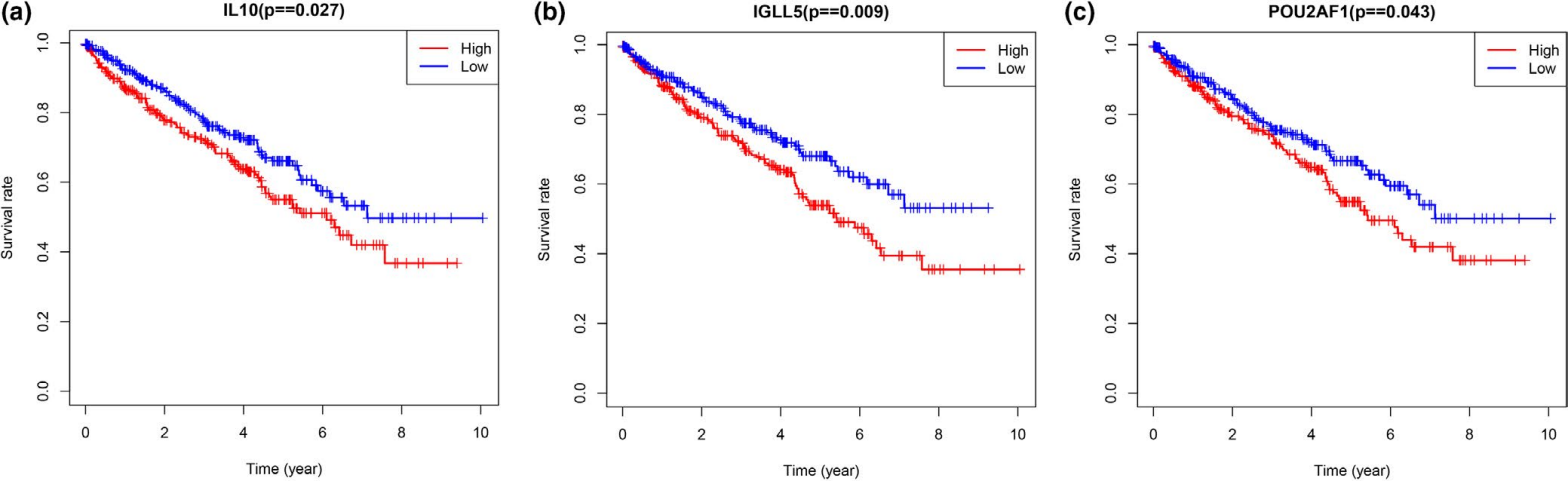
Kaplan–Meier analysis results of hub genes (*p* < .05). Three hub genes were found to be correlated with the prognosis of ccRCC patients: (a) IL10, (b) IGLL5, and (c) POU2AF1

To identify hub genes of the ccRCC microenvironment, we further analyzed the PPI network of DEGs using the degree algorithm in Cytoscape software. Ten hub genes were screened, based on their degree score. The screened hub genes were as follows: *CD19, CD79A*, *IL10*, *IGLL5*, *POU2AF1*, *CCL19*, *AMBP*, *CCL18*, *CCL21*, and *IGJ* (Figure 9a,b and Table 4). A network of the hub genes and their coexpressed genes was analyzed using the cBioPortal database, with a total of six hub genes in this network (Figure 9c). We analyzed the correlation between hub gene expression and overall ccRCC patient survival; the results are shown in Figure 10.

**Table 4:** List of 10 hub genes

Rank	Gene name	Gene ID	Description	Location	Expression	Degree score
1	*CD19*	930	CD19 molecule	Chr16p11.2	Upregulated	19
2	*CD79A*	973	CD79a molecule	Chr19q13.2	Upregulated	13
3	*IL10*	3586	interleukin 10	Chr1q32.1	Upregulated	11
4	*IGLL5*	100423062	immunoglobulin lambda‐like polypeptide 5	Chr22q11.22	Upregulated	10
5	*POU2AF1*	5450	POU class 2 homeobox associating factor 1	Chr11q23.1	Upregulated	10
6	*CCL19*	6363	C‐C motif chemokine ligand 19	Chr9p13.3	Upregulated	8
7	*AMBP*	259	alpha−1‐microglobulin/bikunin precursor	Chr9q32	Downregulated	8
8	*CCL18*	6362	C‐C motif chemokine ligand 18	Chr17q12	Upregulated	7
9	*CCL21*	6366	C‐C motif chemokine ligand 21	Chr9p13.3	Upregulated	7
10	*IGJ (JCHAIN)*	1114	Ig J chain (joining chain of multimeric IgA and IgM)	Chr4q13.3	Upregulated	7

## 4 DISCUSSION

In this study, we used TCGA database to screen tumor microenvironment‐related genes to explore the novel prognostic biomarkers and therapeutic targets of ccRCC. Moreover, we compared the gene expression profiles of 530 patients with high versus low immune scores (or stromal scores) and found 42 genes to be involved in the immune response.

First, we analyzed the relationship between immune/stromal scores and prognosis of ccRCC and found patients with high immune/stromal scores had a worse overall survival. In addition, the immune scores were significantly related to the clinical parameters (gender, grade, pathological stage, T stage, and M stage). These results suggested that the change of tumor microenvironment prominently correlated with the prognosis and development of ccRCC. We then analyzed the gene expression profiles of the patients in the high‐ versus low‐score groups and obtained 95 DEGs, which included 48 upregulated and 47 downregulated genes. Overall survival analysis of these 95 genes resulted in the identification of 43 genes associated with ccRCC patient outcomes. These results showed that tumor microenvironment‐related genes are an important predictor of ccRCC patient prognosis.

Next, we subjected the 95 DEGs to GO and KEGG enrichment analyses. Results of the GO functional analysis indicated that DEGs were mainly enriched in the immune response (BP), extracellular region (CC), and cytokine activity (MF). These results suggest that the DEGs play a vital role in regulating the ccRCC microenvironment, and could influence ccRCC development. Previous studies have suggested that the immune response contributes to tumor progression and drug resistance of various cancers (Astaneh, Dashti, & Esfahani, 2019; Miranda et al., 2019; Pitt et al., 2016), including bladder (Flores‐Martín et al., 2019; Holland et al., 2019), lung (Hays & Bonavida, 2019; Sorich, Rowland, Karapetis, & Hopkins, 2019), and breast cancers (Wagner et al., 2019). The results of the KEGG enrichment analyses suggested that the DEGs were mostly enriched in cytokine‒cytokine receptor interaction, hematopoietic cell lineage, primary immunodeficiency, chemokine signaling pathway, steroid hormone biosynthesis, and intestinal immune network for IgA production. These signaling pathways also play a key role in the ccRCC microenvironment and the progression of ccRCC. Previous studies indicated that the cytokine‒cytokine receptor interaction might participate in the development of glioblastoma (GBM) (Agrawal et al., 2018) and osteosarcoma (Tsukamoto et al., 2012), and modulate the tumor microenvironment of hematopoietic cell lineage. Therefore, regulation of the tumor microenvironment could impact tumor progression (Flores et al., 2018; Xiong et al., 2015). Hematopoietic stem cells, through the myeloid lineage, may act as progenitors for cancer‐associated adipocytes (CAAs) and cancer‐associated fibroblasts (CAFs); these cells could remodel the tumor microenvironment, thereby driving all aspects of tumor progression, including tumor growth and survival, chemoresistance, tumor vascularization, tumor invasion, and tumor cell metastasis (Xiong et al., 2015). Moreover, primary immunodeficiency (Yazdani et al., 2017), chemokine signaling pathway (Meng, Xue, & Chen, 2018; Zhou, Cao, Li, & Zhao, 2018), steroid hormone biosynthesis (Boibessot & Toren, 2018; Hima & Sreeja, 2016), and intestinal immune network for IgA production (Liang et al., 2018) all participate in tumor progression and regulation of the tumor microenvironment. Moreover, we performed module analyses on the constructed PPI network, selected the most significant module, and performed BP analysis of the genes in this module. We found that the genes in the module were primarily enriched in the B‐cell receptor signaling pathway. These results indicate that the development of ccRCC and changes to the ccRCC microenvironment might be related to these biological processes.

Finally, we selected 10 DEGs as hub genes using the degree algorithm. The hub genes are *CD19*, *CD79A*, *IL10*, *IGLL5*, *POU2AF1*, *CCL19*, *AMBP*, *CCL18*, *CCL21,* and *IGJ* (*JCHAIN*). We then analyzed the correlation between these 10 DEGs and the prognosis of ccRCC patients and found that three hub genes (*IL10, IGLL5, and POU2AF1*) were associated with overall patient survival. These three genes are of particular interest. Interleukin‐10 (*IL10*) is produced by immune cells (e.g., macrophages, T lymphocytes, and natural killer cells) and functions as a multifunctional immune‐regulatory cytokine with both immunosuppressive and antiangiogenic effects (Sheikhpour et al., 2018). *IL10* plays an important role in both immune‐mediated diseases and cancer (Geginat et al., 2016; Mannino et al., 2015); Wang et al found that lower serum *IL10* levels were correlated with a better prognosis in cervical cancer patients (Wang et al., 2018). In addition, *IL10* might promote tumor cell proliferation and metastasis through immunosuppression. A previous study has shown that it promotes *IL6* expression and synthesis, which gives rise to cell proliferation via B‐cell lymphoma‐2 (*Bcl‐2*) upregulation that changes the proliferation/apoptosis equivalence toward neoplastic cell proliferation (Sheikhpour et al., 2018). *IGLL5* encodes one of the immunoglobulin lambda‐like polypeptides and plays a significant role in tumor progression. Liang et al reported that fusion of the *IGLL5* gene might promote metastasis of the lymph nodes and play a role in breast cancer development (Liang et al., 2015). Moreover, White et al found that an *IGLL5* mutation was associated with the incidence and progression of multiple myeloma (MM) (White et al., 2018). *POU2AF1* is also an important gene in tumorigenesis and tumor progression. Zhao et al. reported that *POU2AF1* is activated by amplification (or through other mechanisms) and may promote MM progression by accelerating the growth of MM cells through direct transactivation of one of its target genes, *TNFRSF17* (Zhao et al., 2008). Expression dysregulation or *POU2AF1* mutation is also related to the development of chronic lymphocytic leukemia (CLL) (Auer et al., 2005).

The tumor microenvironment plays an important role in the evolution of tumors. For example, in breast cancer, changes in the tumor microenvironment components were considered a key element for cancer development and progression, as well as potential therapeutic targets. Various components of the tumor microenvironment, such as suppressive immune cells, soluble factors, and altered extracellular matrix, act together to impede effective antitumor immunity and promote breast cancer progression and metastasis (Soysal, Tzankov, & Muenst, 2015). In addition, changes in the tumor microenvironment have also been found to be correlated with drug resistance; Mikami et al. (2019) detected upregulated expression of PD‐1 and PD‐L1 in tumor‐infiltrating immune cells (TIIC) in the tumor microenvironment, related to the resistance of vascular endothelial growth factor‐tyrosine kinase inhibitors (VEGF‐TKIs).

In the present study, we examined how patterns of gene expression in the tumor microenvironment influenced components of the microenvironment and the prognosis of ccRCC patients. Our results provide data that will support further explorations of the complex interactions between the tumor and its environment in ccRCC. Inevitably, our study had a few limitations. First, we used data from public databases that were not verified in prospective clinical trials. Second, the functions of the DEGs that impact the development of ccRCC need to be investigated further through in vivo and in vitro experiments.

## 5 CONCLUSION

In summary, our study revealed correlations between the ccRCC microenvironment and patient prognosis, and also between gene expression in the microenvironment and overall survival of patients. These findings increase the understanding of the mechanisms through which gene expression affects the prognosis and development of ccRCC through the tumor microenvironment. This study also screened several candidate genes and biological pathways that may contribute to the search for biomarkers and therapeutic targets of ccRCC. However, further experiments are necessary to probe the biological function of these genes in ccRCC.

## CONFLICT OF INTERESTS

The authors declare that they have no competing interests.

## AUTHOR CONTRIBUTIONS

Bangbei Wan, Yuan Huang, and Cai Lv conceived and designed the study. Bangbei Wan and Bo Liu revised the images and performed data analysis. Bangbei Wan and Cai Lv wrote and revised the manuscript.

## ETHICAL STATEMENT

Our study did not require an ethical board approval because it did not contain human or animal trials.
